# The contribution of physical working conditions to sickness absence of varying length among employees with and without common mental disorders

**DOI:** 10.1177/1403494820901411

**Published:** 2020-01-21

**Authors:** Jaana I. Halonen, Tea Lallukka, Tero Kujanpää, Jouni Lahti, Noora Kanerva, Olli Pietiläinen, Ossi Rahkonen, Eero Lahelma, Minna Mänty

**Affiliations:** 1 Department of Public Health, University of Helsinki, Helsinki, Finland; 2 Finnish Institute of Occupational Health, Helsinki, Finland; 3 Center for Life Course Health Research, Faculty of Medicine, University of Oulu, Finland; 4 City of Vantaa, Vantaa, Finland

**Keywords:** Computer work, hazardous exposure, physical work, common mental disorder, sickness absence, shift work

## Abstract

**Aims:** The aim was to examine whether the contribution of physical work exposures to the risk of sickness absence (SA) is different between those with and without common mental disorders (CMD). **Methods:** We used questionnaire data on four work exposures and CMD from 6159 participants of the Helsinki Health Study cohort with 12,458 observations from three surveys (2000–2002, 2007 and 2012). We formed combination exposures for the work exposures (hazardous exposures, physical workload, computer and shift work) with CMD. Associations with SA of different length were examined with negative binomial regression models. **Results:** We observed stronger associations for CMD with SA than for the individual work exposures. The strength of the associations for hazardous exposures and physical workload increased with length of SA, especially when the participant also had CMD. The strongest associations for the combined exposures were observed for SA ⩾15 days, the rate ratios being 2.63 (95% CI 2.27–3.05) among those with hazardous exposure and CMD, and 3.37 (95% CI 2.93−3.88) among those with heavy physical workload and CMD. **Conclusions: Employees with hazardous exposures or physical workload combined with CMD were at the highest risk of SA compared with those without these exposures or with only one exposure.**

## Background

Throughout the Organisation for Economic Co-operation and Development (OECD) countries, work disability is a major social and economic problem [1]. Many psychosocial and physical characteristics of work as well as shift work have been linked to an increased risk of work disability, namely, sickness absence (SA) [2-5]. In addition to the work-related factors, mental disorders are a major cause of work disability [1,6,7]. Even the less severe forms of mental disorders such as common mental disorders (CMD), have been associated with a higher risk of SA [8,9]. To date, there are few studies on the combined associations of work exposures and CMD, and these have focused on the associations for psychosocial work exposures and mental health with intermediate or long-term SA [10,11]. However, we are not aware of studies that have examined how associations between different physical working conditions or shift work and risk of SA vary by an individual’s state of mental health, and whether these associations vary by the severity of disability. It is important to know whether people with CMD are more vulnerable to different stressors in working life than those without CMD, as targeted support for work ability and work modifications can then be planned.

To fill these gaps in evidence we examined whether associations between physical work exposures and SA vary according to mental health status. We assessed associations for four combinations of physical work exposures (including shift work) and CMD with short-term, intermediate, and long-term SA. The physical work exposures included hazardous exposure (chemicals, dust and noise), physical workload (e.g. repetitive trunk rotation, lifting and carrying), computer work (working with computer and mouse, and sitting), and shift work. Shift work was included as it is an indicator of the strenuousness of work and can affect circadian rhythm, tiredness and consequently, work ability [12,13]. These analyses increase the level of our understanding regarding the roles of mental health and physical work in SA. We hypothesised that the physical work exposures are more strongly associated with SA among those who also experienced CMD when compared with those not reporting these stressors. Furthermore, we hypothesised that the associations for the combinations of physical work exposures and CMD vary by the length of SA.

## Methods

We used data from the Helsinki Health Study, which is a longitudinal cohort study on health and well-being of ageing municipal employees covering a large scale of different occupations (e.g. healthcare, social welfare, education, culture, public transport). All the employees of the City of Helsinki, Finland, aged 40, 45, 50, 55, and 60 years in 2000, 2001 and 2002 were asked to participate in a mail survey, which included questions on their health, health behaviours and working conditions. A total of 8960 employees responded (response rate 67%) at Phase 1 between 2000 and 2002. First follow-up, i.e. Phase 2 (response rate 83%), was collected in 2007, and Phase 3 in 2012 (response rate 78%). The survey data have been prospectively linked with employers’ personnel register data on SA for all those who gave written informed consent (78%). Non-response analyses have suggested the data to be broadly representative of the target population [14,15]; for example, many occupations are female dominated, including teachers, nurses and healthcare assistants. However, men, manual workers, and those with long SA were somewhat overrepresented among the non-respondents.

**Figure 1.**
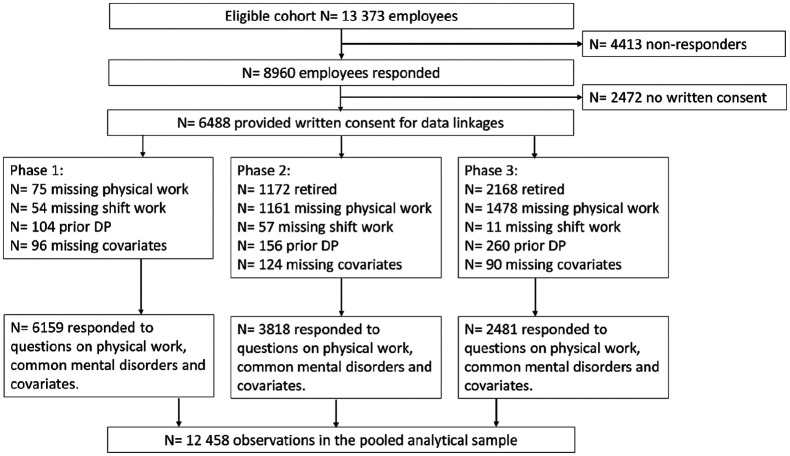
Flow chart of the sample selection. DP: Disability pension.

For this study, we included participants who responded to the mailed surveys including items regarding four physical work exposures, CMD and covariates in each Phase. After excluding those with any missing data we included 6159 observations from the Phase 1, 3818 observations from Phase 2, and 2481 observations from Phase 3. Thus, the pooled data from the three surveys resulted in 12,458 observations from 6159 individuals (Figure 1). For all observations, the follow-up period for SA started from the date when the survey was filled in and lasted for 1 year. Ethics approval was provided by the Ethics Committees of the Department of Public Health, University of Helsinki and the health authorities of the City of Helsinki, Finland.

### Exposures

All exposure variables, i.e. four physical work exposures and CMD, were identically requested in the three surveys (2000–2002, 2007 and 2012).

#### Physical work exposures

The surveys included an 18-item inventory regarding different physical work exposures [16]. Of the 18 four-category items (response options: not at all; yes, but does not affect work; yes and affects work negatively to some degree; yes and affects work negatively a lot), three broad factors were obtained using a factor analysis with polychoric correlation matrix [3]. The first factor comprised items related to *hazardous exposure* in the work environments such as hazardous chemicals, dust and noise; the second items related to *physical workload*, such as repetitive trunk rotation, lifting and carrying; and the third factor, *computer work*, comprised working with computer and mouse, and sitting. Loadings for all three factors were divided into quartiles and the highest quartile indicated exposure to each factor [17]. Shift work was investigated in all surveys with six response alternatives, and it was dichotomised into ‘normal day work’ vs. ‘shift work’. Shift work included working in day shifts without night work, three-shift work including night work, and night work only. The proportion of participants with night work only was very low (1.3%).

#### Common mental disorders

CMD were measured with the General Health Questionnaire (GHQ-12) often used in occupational studies, which has shown comparable results to the original 60-item questionnaire [18]. It has also shown high reliability and good validity in relation to diagnosed affective disorders [18-20]. CMD includes information on general and context-free affective and non-psychotic problems in the past few weeks, such as symptoms of depression, anxiety and poor self-esteem. The GHQ-12 scores range from 0 to 12. To identify employees with CMD we used a cut-off point 3 that has shown to be a valid threshold for this scale [20].

### Outcomes

Many employers in Finland, like the City of Helsinki, allow SA of 1–3 days based on self-certification, i.e. without a medical certificate. The employer will continue to pay full salary – depending on the length of employment – up to 2 months. The Social Insurance Institution (SII) will pay sickness allowance after 10 work days of absence up to 300 work days, if necessary. SA data for this study were obtained from the employer’s personnel register. We used prevalence of SA of any length as well as SA by length: self-certified short-term (1–3 days), and medically certified intermediate (4–14 days) and long-term (⩾15 days) SA spells during a 1-year follow-up from the date of survey response. This means that after each survey, there was a new follow-up period for SA. The causes of the SA spells were not available from the employers’ register.

### Covariates

Age, gender, marital status, education, body mass index (BMI, based on weight and height), smoking, binge drinking, and physical chronic diseases were self-reported in each survey. These were considered as covariates as they have shown associations with mental health and work disability [11,21]. Education was treated as three-class variable: low = vocational school or less; intermediate = high school or college; high = university degree. Marital status was also categorised into three classes: single, married/cohabiting and divorced/widowed. BMI was calculated from self-reported weight and height, as weight in kilograms divided by height in metres squared and categorised as: non-obese = BMI <30 kg/m^2^, and obese BMI ⩾30 kg/m^2^. Smoking was treated as a dichotomous variable: non-smoker vs. current smoker. Binge drinking was asked as the frequency of having consumed more than six units of alcohol at one occasion, and it was dichotomised as: no bingeing = less than once a month for women and less than once a week for men, and bingeing = more than once a month for women and once a week for men. Different cut-offs were used due to a large gender difference in drinking habits in this study population [22]. Self-reported physician-diagnosed chronic diseases included cardiovascular diseases (angina pectoris, heart attack, cerebral haemorrhage), diabetes and cancer. Chronic diseases were categorised as: 0, 1 or ⩾2 [23].

Exposure to heavy physical workload may lead to changes in leisure-time physical activity [24], and poorer mental health has been associated with greater physical inactivity [25]. As vigorous physical activity has further been linked to decreased risk of SA [26], physical activity can act as a mediator between physical work exposures and SA. Thus, we did not include physical inactivity as a covariate into the main analyses, but ran additional analyses adjusting for it. Average weekly hours of physical activity during leisure time or commuting within the previous 12 months was requested at four levels of intensity: walking, brisk walking, jogging and running, or their equivalent activities. For each level of intensity, five response alternatives were available from 0 to 4 or more hours/week. We calculated metabolic equivalent of task (MET) hours per week by multiplying the time spent in physical activity with the MET value of each intensity level and adding these up [27]. Participants with weekly MET hours <14 were classified as physically inactive [26].

### Statistical analyses

For the analyses, we formed four exposure variables combining the different work exposures and CMD. Each of the four variables included dichotomised work exposure and dichotomised CMD that were categorised into four classes: ‘neither’, ‘work exposure only’, ‘CMD only’ and ‘both’. The variables were named as: ‘*hazardous exposures/CMD*’, ‘*physical workload/CMD*’, ‘*computer work/CMD*’ and ‘*shift work/CMD*’.

The associations between the exposure combinations and the number of total SA spells, spells lasting 1–3 days, 4–14 days and ⩾15 days during a 1-year follow-up were separately assessed using generalised estimating equations. This method accounts for within-person correlation from those participants who contributed observations to the pooled data from more than one survey phase (proc genmod in SAS). We used negative binomial distribution in the regression models that were first adjusted for age at Phase 1 and gender. Data for further adjustments; marital status, education, smoking, binge drinking, obesity and chronic disease were from the survey where the exposure was reported. We conducted all analyses as complete case analyses where all participants with missing data on exposures or covariates were excluded. Results are presented as rate ratios (RRs) with 95% confidence intervals (CI). As sensitivity analyses we ran the models with additional adjustment for physical inactivity. All statistical analyses were performed using SAS version 9.4 (SAS Institute, Inc., Cary, North Carolina).

## Results

Of the study population 80% were women, and the mean age at the start of the follow-up was 51.8 (SD = 6.3) years. Other descriptive statistics for all participants and by CMD at Phase 1 are presented in Table I. Of the total analysis sample 21% were physically inactive, and the corresponding percentages were 28% for those with CMD, and 19% for those without CMD. Prevalence of the SA spells per 100 person-years by the four exposure variables is shown in Table II. Mean follow-up time for any SA spell was 350 (SD = 54) days.

**Table I.** Study population characteristics by common mental disorders (CMD) at Phase 1.

Variable	All (*n*=6159)		CMD (*n*=1504)		No CMD (*n*=4655)		Difference CMD vs. No CMD
	*n*	%	*n*	%	*n*	%	*p*-value
Women	4826	78	1193	79	3633	78	0.296
Marital status							0.012
Single	766	12	197	13	569	12	
Married/cohabiting	4355	71	1020	68	3335	72	
Divorced/widowed	1038	17	287	19	751	16	
Education							0.479
Low	2424	39	572	38	1852	40	
Intermediate	2016	33	502	33	1514	32	
High	1719	28	430	29	1289	28	
Smoker	1416	23	396	26	1020	22	0.0004
Binge drinker	2924	47	774	51	2150	46	0.0004
Obese	896	15	247	16	659	14	0.018
Chronic disease							0.017
0	5416	88	1292	86	4124	88	
1	687	11	194	13	493	11	
⩾2	56	1	18	1	38	1	
Physically inactive	1316	21	418	28	898	19	<0.0001
Hazardous exposure	1508	25	461	31	1047	22	<0.0001
Physical workload	1499	24	487	32	1012	22	<0.0001
Computer work	1543	25	511	34	1032	22	<0.0001
Shift work	1404	23	357	24	1047	22	0.317

**Table II.** Sickness absence spells/100 person-years by length of absence and exposure.

**Exposure**	Sickness absence (SA)					
	n observations	All SA	1-3 days	4-14 days		≥15 days
Hazardous exposure / CMD						
neither	7353	190	131	43	13	
work exposure only	2142	218	134	61	23	
CMD only	2051	243	150	62	31	
both	911	312	177	89	46	
Physical workload / CMD						
neither	7442	185	130	41	14	
work exposure only	2053	238	137	69	32	
CMD only	2010	227	148	52	27	
both	952	343	181	108	54	
Computer work / CMD						
neither	7378	201	133	49	19	
work exposure only	2117	179	124	39	16	
CMD only	1941	276	161	76	39	
both	1021	243	154	59	30	
Shift work / CMD						
neither	7536	197	135	44	18	
work exposure only	1959	194	119	56	18	
CMD only	2294	259	157	68	35	
both	668	282	164	79	39	

**Figure 2.**
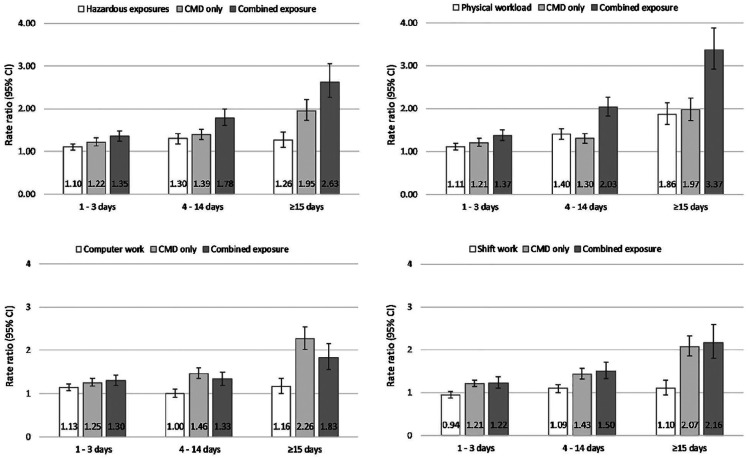
Rate ratios (95% confidence intervals) for sickness absence of varying length by physical work exposures and common mental disorders (CMD). Note: Models adjusted for gender, age, marital status, education, smoking, binge drinking, obesity, and chronic disease. CMD: common mental disorders.

Table III shows the age and gender adjusted RRs for SA spells of varying length by the four exposure variables. Adjustment for the covariates slightly attenuated the effect estimates. The fully adjusted RRs are presented in Figure 2 and the corresponding numerical values are provided in Supplemental Table 1. Of the work exposures alone, computer work was associated with self-certified SA spells of 1–3 days (RR 1.13, 95% CI 1.06–1.22) in the fully adjusted model, but it was not associated with the longer medically certified SA spells. Hazardous exposures were also weakly associated with the shortest SA spells, but stronger associations were observed for the SA spells lasting 4–14 days (RR 1.30, 95% CI 1.18–1.42) and ⩾15 days (RR 1.26, 95% CI 1.09–1.46). An increasing pattern was observed for heavy physical workload: RR was 1.40 (95% CI 1.28–1.53) for SA spells 4–14 days, and 1.86 (95% CI 1.63–2.13) for SA spells ⩾15 days. Reporting shift work alone was not associated with SA spells. In general, effect estimates for CMD alone were larger than those for the work exposures alone.

**Table III.** Rate ratios (RR, 95% confidence intervals) for sickness absence of varying length by the combined exposures.

**Exposure**	All			1–3 days			4–14 days			⩾15 days		
	RR*	95% CI		RR*	95% CI		RR*	95% CI		RR*	95% CI	
Hazardous exposure / CMD												
neither	1			1			1			1		
work exposure only	1.22	1.15	1.29	1.13	1.05	1.21	1.40	1.28	1.53	1.39	1.21	1.61
CMD only	1.33	1.25	1.42	1.24	1.15	1.33	1.39	1.27	1.52	1.94	1.72	2.20
both	1.71	1.58	1.84	1.45	1.32	1.59	1.97	1.77	2.21	3.04	2.62	3.54
Physical workload / CMD												
neither	1			1			1			1		
work exposure only	1.34	1.27	1.43	1.16	1.08	1.25	1.62	1.49	1.77	2.18	1.91	2.48
CMD only	1.30	1.22	1.38	1.24	1.15	1.33	1.29	1.18	1.41	1.96	1.72	2.23
both	1.88	1.74	2.02	1.48	1.35	1.62	2.37	2.12	2.65	4.05	3.52	4.65
Computer work / CMD												
neither	1			1			1			1		
work exposure only	1.02	0.96	1.09	1.08	1.01	1.16	0.87	0.79	0.95	1.00	0.87	1.16
CMD only	1.42	1.34	1.51	1.29	1.20	1.38	1.48	1.35	1.61	2.31	2.05	2.61
both	1.31	1.22	1.41	1.30	1.19	1.42	1.22	1.08	1.37	1.68	1.42	1.98
Shift work / CMD												
neither	1			1			1			1		
work exposure only	1.10	1.03	1.18	1.02	0.94	1.11	1.28	1.16	1.41	1.29	1.12	1.50
CMD only	1.37	1.29	1.45	1.25	1.17	1.34	1.44	1.32	1.57	2.11	1.88	2.37
both	1.54	1.41	1.69	1.34	1.20	1.50	1.77	1.55	2.02	2.59	2.16	3.11

* Models adjusted for gender and age.

The combination of hazardous exposures and CMD as well as physical workload and CMD together resulted in the highest RRs, particularly regarding the longer, medically certified SA spells. Hazardous exposures and CMD resulted in a RR of 1.78 (95% CI 1.60–1.99) for the 4–14-day SA, and RR of 2.63 (95% CI 2.27–3.05) for SA spells of ⩾15 days. The corresponding RRs for physical workload and CMD were 2.03 (95% CI 1.82–2.27) and 3.37 (95% CI 2.93–3.88). The findings suggest joint contribution of these physical work exposures and CMD on the long-term SA as indicated by the non-overlapping confidence intervals when compared with those of the individual exposures. Additional adjustment for physical inactivity had a minor effect on the estimates in the fully adjusted models (Supplemental Table 2).

## Discussion

Of the four examined work exposures, heavy physical workload and hazardous exposure alone had the strongest associations with all-cause SA, particularly the medically certified SA spells lasting 4 days or more. Combined exposure to heavy physical workload or hazardous exposure and self-reported CMD resulted in even stronger associations with SA spells, the associations being the stronger the longer term the absence spell was. Computer work alone predicted weakly the self-certified short-term SA spells (1–3 days) and having CMD did not strengthen this association significantly. Shift work was not associated with SA.

We observed associations for physical workload and hazardous exposures with SA that are in line with the existing literature [2-4], although in the earlier studies the role of CMD was not considered. Joint associations, i.e. reporting combination of physical workload and CMD, or hazardous exposure and CMD, were considerably stronger for the medically certified SA spells than either of the exposures alone. This suggests that those with CMD and work tasks including working in uncomfortable postures, repetitive trunk rotation and movements, standing, lifting and carrying form a particularly vulnerable group of employees. It may be, however, that jobs including physical tasks are such where any work disability entails SA. Nurses, for example, may have physical strain in their work and they are advised to take sick leave even for mild flu to not to expose the patients. Construction workers, then, may be advised to recover longer periods from somatic diseases than those with sedentary work.

Our findings also suggest that work including use of computer as well as sitting is associated mainly with the less severe health problems requiring SA lasting only for 1–3 days. A prior study examining different lengths of SA neither reported an association between computer work and SA exceeding 4 days [3]. The association for combined exposure to computer work and CMD with short-term SA was only slightly stronger than that for CMD alone. Regarding the longer SA spells, the associations for CMD alone were even stronger than those for the combined exposure. This suggests that computer work alone or with CMD contribute little to SA in these data. However, computer work may also be such that people are working even if they have some degree of work disability, as for example remote work from home may be possible, and this may have masked some associations. In line with prior findings [5], shift work alone was not associated with the longer term SA, and the associations for short-term SA were also weak. As the associations between CMD alone and SA were nearly as strong as the associations for the combined exposure to shift work and CMD, it is likely that the joint associations were mostly due to the CMD.

One of the strengths of this study is the longitudinal design, a large employee cohort including a broad range of different occupations and employers’ register data on employees’ SA spells. Though cause-specific data were not available, these data included the scarcely examined short-term SA spells for which the absence benefits are covered by the employer and that are not available from national registers. The cut-off for short SA was based on the length of self-certification period, whereas the cut-off differentiating intermediate and long absence spells, while used in prior studies [8,26], was more arbitrary. Two weeks, indicated by the intermediate absence, can be thought to catch, for example, prolonged flu, and it is also in line with the lower limit of absence spells covered by the SII (i.e. 10 weekdays). The longer absence spells would include more severe health conditions and injuries. We used self-administered measures for the work exposures and CMD, and thus reporting bias may have led to under- or overestimation of the actual exposures. To define CMD, we used GHQ-12 questionnaire that covers symptoms of minor mental health problems. It has been shown to be a reliable and well-validated measure suitable for use in general and employee populations [28], although it does not distinguish between more severe conditions such as depression or anxiety. We cannot rule out non-response and healthy worker effect that might lead to underestimation of the findings, although previous non-response analysis on these data has shown that these data represent well the target population [14,15]. However, the generalisability of our findings to employment sectors other than public sector and to general populations may be limited, as we used data only from the Finnish public sector. Finally, although this is a longitudinal study, we cannot interpret causality regarding the observed associations as the participants are likely to have had physical work exposures and SA spells already before the survey measurements used for these analyses [29].

In summary, our findings suggest that heavy physical workload and hazardous exposures had the stronger associations with SA the longer the SA spell was. Importantly, if there was combined exposure to hazardous exposures and CMD, or heavy physical workload and CMD, the likelihood of longer term medically certified SA increased substantially. Thus, it would be important to recognise and support those employees who have high physical workload and suffer from CMD, and to plan strategies on preventing their SA in the most efficient way. These strategies could include possibilities to make targeted work modifications that ease the workload, or work rotation, for example.

## Supplemental Material

SJP901411_Supplemental_material – Supplemental material for The contribution of physical working conditions to sickness absence of varying length among employees with and without common mental disorders Click here for additional data file. Supplemental material, SJP901411_Supplemental_material for The contribution of physical working conditions to sickness absence of varying length among employees with and without common mental disorders by Jaana I. Halonen, Tea Lallukka, Tero Kujanpää, Jouni Lahti, Noora Kanerva, Olli Pietiläinen, Ossi Rahkonen, Eero Lahelma and Minna Mänty in Scandinavian Journal of Public Health
