# Supplementary material for: The contribution of physical working conditions to sickness absence of varying length among employees with and without common mental disorders
Source: Scand J Public Health. 2020 Jan 21;49(2):141–8. doi: 10.1177/1403494820901411 (PMC7917561; doi:10.1177/1403494820901411)
Supplement: SJP901411_Supplemental_material – Supplemental material for The contribution of physical working conditions to sickness absence of varying length among employees with and without common mental disorders [file SJP901411_Supplemental_material.pdf]

Supplemental material

The contribution of physical working conditions to sickness absence of varying length among employees with and without common mental disorders

Jaana I. Halonen, Tea Lallukka, Tero Kujanpää, Jouni Lahti, Noora Kanerva, Olli Pietiläinen, Ossi Rahkonen, Eero Lahelma, Minna Mänty

Supplemental Table 1. Fully adjusted rate ratios* (RR, 95% confidence intervals) for sickness absence of varying length by categories of the exposure variables.

	All			1-3 days			4-14 days			≥15 days		
**Exposure**	RR	95% CI		RR	95% CI		RR	95% CI		RR	95% CI	
Hazardous exposure / CMD												
neither	1			1			1			1		
work exposure only	1.16	1.10	1.23	1.10	1.03	1.18	1.30	1.18	1.42	1.26	1.09	1.46
CMD only	1.31	1.24	1.40	1.22	1.13	1.31	1.39	1.28	1.52	1.95	1.73	2.21
both	1.57	1.46	1.69	1.35	1.24	1.48	1.78	1.60	1.99	2.63	2.27	3.05
Physical workload/ CMD												
neither	1			1			1			1		
work exposure only	1.23	1.16	1.31	1.11	1.03	1.19	1.40	1.28	1.53	1.86	1.63	2.13
CMD only	1.28	1.21	1.36	1.21	1.12	1.30	1.30	1.19	1.42	1.97	1.73	2.24
both	1.69	1.57	1.82	1.37	1.25	1.50	2.03	1.82	2.27	3.37	2.93	3.88
Computer work / CMD												
neither	1			1			1			1		
work exposure only	1.11	1.05	1.18	1.13	1.06	1.22	1.00	0.91	1.10	1.16	1.00	1.35
CMD only	1.39	1.31	1.48	1.25	1.17	1.34	1.46	1.34	1.59	2.26	2.01	2.54
both	1.35	1.25	1.45	1.30	1.19	1.42	1.33	1.18	1.49	1.83	1.56	2.15
Shift work / CMD												
neither	1			1			1			1		
work exposure only	0.99	0.92	1.06	0.94	0.87	1.02	1.09	0.99	1.19	1.10	0.94	1.28
CMD only	1.33	1.26	1.41	1.21	1.13	1.29	1.43	1.31	1.56	2.07	1.85	2.33
both	1.37	1.25	1.50	1.22	1.09	1.36	1.50	1.32	1.70	2.16	1.81	2.59

* Models adjusted for sex, age, marital status, education, smoking, binge drinking, obesity, and chronic disease

Supplemental Table 2. Physical inactivity adjusted rate ratios* (RR, 95% confidence intervals) for sickness absence of varying length by categories of the exposure variables.

	All			1-3 days			4-14 days			≥15 days		
**Exposure**	RR	95% CI		RR	95% CI		RR	95% CI		RR	95% CI	
Hazardous exposure / CMD												
neither	1			1			1			1		
work exposure only	1.17	1.10	1.25	1.12	1.04	1.20	1.30	1.18	1.43	1.24	1.07	1.44
CMD only	1.31	1.23	1.40	1.21	1.12	1.30	1.40	1.27	1.54	1.91	1.68	2.18
both	1.56	1.45	1.69	1.35	1.23	1.49	1.75	1.55	1.96	2.61	2.24	3.05
Physical workload/ CMD												
neither	1			1			1			1		
work exposure only	1.24	1.16	1.32	1.11	1.03	1.20	1.40	1.28	1.54	1.86	1.62	2.15
CMD only	1.28	1.20	1.37	1.21	1.12	1.31	1.30	1.18	1.43	1.95	1.70	2.24
both	1.66	1.53	1.80	1.34	1.22	1.47	2.00	1.77	2.25	3.32	2.86	3.86
Computer work / CMD												
neither	1			1			1			1		
work exposure only	1.10	1.03	1.18	1.12	1.04	1.21	1.02	0.92	1.12	1.15	0.98	1.34
CMD only	1.38	1.29	1.47	1.24	1.15	1.34	1.45	1.33	1.59	2.20	1.94	2.50
both	1.33	1.23	1.44	1.27	1.16	1.40	1.33	1.17	1.50	1.86	1.57	2.20
Shift work / CMD												
neither	1			1			1			1		
work exposure only	0.99	0.93	1.07	0.94	0.86	1.02	1.11	1.01	1.23	1.08	0.92	1.27
CMD only	1.33	1.25	1.41	1.21	1.12	1.30	1.43	1.31	1.57	2.05	1.82	2.32
both	1.34	1.22	1.48	1.19	1.06	1.34	1.49	1.30	1.71	2.10	1.74	2.54

* Models adjusted for sex, age, marital status, education, smoking, binge drinking, obesity, chronic disease and physical inactivity
